# Supplementary material for: A UMLS-based spell checker for natural language processing in vaccine safety
Source: BMC Med Inform Decis Mak. 2007 Feb 12;7:3. doi: 10.1186/1472-6947-7-3 (PMC1805499; doi:10.1186/1472-6947-7-3)
Supplement: Additional file 13 — RAP application directory. Sets up directory for RAP (RDF application for PHP) source code [file 1472-6947-7-3-S13.gz › rap/doc/tests/Test_Serializer_Options.htm]

Test Serializer Options


### RDF API for PHP V0.9.1

# Test: Serializer Options

This example is part of the RAP - Rdf API for PHP documentation.

Used document: example1.rdf

`<?xml version="1.0"?>  
<rdf:RDF   
xml:base="http://www.w3.org/Home/Lassila.rdf#"  
xmlns:rdf="http://www.w3.org/1999/02/22-rdf-syntax-ns#"  
xmlns:dc="http://purl.org/dc/elements/1.1/"  
xmlns:ex="http://example.org/stuff/1.0/"  
xmlns:s="http://description.org/schema/">  
<rdf:Description rdf:about="http://www.w3.org/Home/Lassila">  
<s:Creator>  
<rdf:Description rdf:nodeID="b85740">  
<rdf:type rdf:resource="http://description.org/schema/Person"/>  
<ex:Name rdf:datatype="http://www.w3.org/TR/xmlschema-2#string">Ora
Lassila</ex:Name>  
<ex:Email>lassila@w3.org</ex:Email>  
</rdf:Description>  
</s:Creator>  
</rdf:Description>  
</rdf:RDF>`

  
  

### 1. Load and show document

`// Include RAP  
define("RDFAPI_INCLUDE_DIR", "./../api/");  
include(RDFAPI_INCLUDE_DIR . "RDFAPI.php");  
include(RDFAPI_INCLUDE_DIR . "PACKAGE_SYNTAX_RDF");`

`// Filename of a RDF document  
$base="example1.rdf";`

`// Create a new MemModel  
$model = ModelFactory::getDefaultModel();`

`// Load and parse document  
$model->load($base);`

`// Output model as HTML table  
$model->writeAsHtmlTable();`

**Output:**

|  |  |  |  |
| --- | --- | --- | --- |
| **Base URI:** http://www.w3.org/Home/Lassila.rdf# | | | **Size:** 4 |
| **No.** | **Subject** | **Predicate** | **Object** |
| 1. | Resource: http://www.w3.org/Home/Lassila | Resource: http://description.org/schema/Creator | Blank Node: b85740 |
| 2. | Blank Node: b85740 | RDF Node: http://www.w3.org/1999/02/22-rdf-syntax-ns#type | Resource: http://description.org/schema/Person |
| 3. | Blank Node: b85740 | Resource: http://example.org/stuff/1.0/Name | Literal: Ora Lassila **(rdf:datatype="http://www.w3.org/TR/xmlschema-2#string")** |
| 4. | Blank Node: b85740 | Resource: http://example.org/stuff/1.0/Email | Literal: lassila@w3.org |

### 2. Create Serializer and serialize model to RDF with default configuration

`// Create Serializer and serialize model
to RDF with default configuration  
$ser = new RDFSerializer();  
$rdf =& $ser->serialize($model);  
echo "<p><textarea cols='110' rows='20'>" .
$rdf . "</textarea>";`

**Output:**

<?xml version='1.0' encoding='UTF-8'?>
<rdf:RDF
xml:base="http://www.w3.org/Home/Lassila.rdf#"
xmlns:rdf="http://www.w3.org/1999/02/22-rdf-syntax-ns#"
xmlns:rdfs="http://www.w3.org/2000/01/rdf-schema#"
xmlns:ns1="http://description.org/schema/"
xmlns:ns2="http://example.org/stuff/1.0/">
<ns1:Person rdf:nodeID="b85740">
<ns2:Email>lassila@w3.org</ns2:Email>
<ns2:Name rdf:datatype="http://www.w3.org/TR/xmlschema-2#string">Ora Lassila</ns2:Name>
</ns1:Person>
<rdf:Description rdf:about="http://www.w3.org/Home/Lassila">
<ns1:Creator rdf:nodeID="b85740"/>
</rdf:Description>
</rdf:RDF>

### 3. Serialize model to RDF using attributes

`// Serialize model to RDF using attributes  
$ser->configUseAttributes(TRUE);  
$rdf =& $ser->serialize($model);  
echo "<p><textarea cols='110' rows='20'>" .
$rdf . "</textarea>";  
$ser->configUseAttributes(FALSE);`

**Output:**

<?xml version='1.0' encoding='UTF-8'?>
<rdf:RDF
xml:base="http://www.w3.org/Home/Lassila.rdf#"
xmlns:rdf="http://www.w3.org/1999/02/22-rdf-syntax-ns#"
xmlns:rdfs="http://www.w3.org/2000/01/rdf-schema#"
xmlns:ns1="http://description.org/schema/"
xmlns:ns2="http://example.org/stuff/1.0/">
<ns1:Person rdf:nodeID="b85740"
ns2:Email="lassila@w3.org">
<ns2:Name rdf:datatype="http://www.w3.org/TR/xmlschema-2#string">Ora Lassila</ns2:Name>
</ns1:Person>
<rdf:Description rdf:about="http://www.w3.org/Home/Lassila">
<ns1:Creator rdf:nodeID="b85740"/>
</rdf:Description>
</rdf:RDF>

### 4. Serialize model to RDF using entities

`// Serialize model to RDF using entities  
$ser->configUseEntities(TRUE);  
$rdf =& $ser->serialize($model);  
echo "<p><textarea cols='110' rows='30'>" .
$rdf . "</textarea>";  
$ser->configUseEntities(FALSE);`

**Output:**

<?xml version='1.0' encoding='UTF-8'?>
<!DOCTYPE rdf:RDF [
<!ENTITY rdf 'http://www.w3.org/1999/02/22-rdf-syntax-ns#'>
<!ENTITY rdfs 'http://www.w3.org/2000/01/rdf-schema#'>
<!ENTITY ns1 'http://www.w3.org/Home/'>
<!ENTITY ns2 ''>
<!ENTITY ns3 'http://description.org/schema/'>
<!ENTITY ns4 'http://example.org/stuff/1.0/'>
]>
<rdf:RDF
xml:base="http://www.w3.org/Home/Lassila.rdf#"
xmlns:rdf="&rdf"
xmlns:rdfs="&rdfs"
xmlns:ns1="&ns1"
xmlns:ns2="&ns2"
xmlns:ns3="&ns3"
xmlns:ns4="&ns4">
<ns3:Person rdf:nodeID="b85740">
<ns4:Email>lassila@w3.org</ns4:Email>
<ns4:Name rdf:datatype="http://www.w3.org/TR/xmlschema-2#string">Ora Lassila</ns4:Name>
</ns3:Person>
<rdf:Description rdf:about="&ns1Lassila">
<ns3:Creator rdf:nodeID="b85740"/>
</rdf:Description>
</rdf:RDF>
